# Validation of a Model Predicting That Physical Activities Improve Health-Related Quality of Life in Older Japanese Adults with Pain, Dysesthesia, and Kinesiophobia after Lumbar Surgery: Structural Equation Modeling

**DOI:** 10.1155/2022/4147497

**Published:** 2022-07-16

**Authors:** Daisuke Higuchi, Yuta Watanabe, Yu Kondo, Takahiro Miki

**Affiliations:** ^1^Takasaki University of Health and Welfare, Takasaki, Japan; ^2^Harunaso Hospital, Takasaki, Japan; ^3^Sapporo Maruyama Orthopedic Hospital, Sapporo, Japan

## Abstract

**Objectives:**

This study assessed the validity of a hypothesized model predicting that physical activity improves health-related quality of life (HRQOL) in older Japanese adults with pain, dysesthesia, and kinesiophobia following lumbar surgery.

**Methods:**

We included 431 elderly patients who underwent surgery for lumbar spinal stenosis at two hospitals. The frequency of physical activity, pain, dysesthesia, kinesiophobia (somatic focus and activity avoidance), and HRQOL were investigated using a questionnaire. Missing values were complemented by the stochastic regression imputation. We constructed the following model. (i) physical activity affects pain, dysesthesia, and kinesiophobia. (ii) pain, dysesthesia, and kinesiophobia separately affect HRQOL. This hypothetical model was tested by structural equation modeling. The model was improved based on a modified index.

**Results:**

Of the 431 respondents, 297 (median age 72 years, range 65–91 years; 158 men and 139 women) were analyzed (68.9%). The fit of the model improved based on the modification index and was acceptable comparative fit index, 0.948; Tucker–Lewis index, 0.919; root mean square error of approximation, 0.048 (90% confidence interval, 0.026–0.069), and standardized root mean square residual (0.046). The paths by which physical activities reduced pain or dysesthesia (standardized pass coefficients, −0.406) and somatic focus (−0.301) and consequently improved HRQOL were significant (pain/dysesthesia, −0.684; somatic focus, −0.218). *Discussion*. Our hypothesized model predicting that physical activity improves HRQOL in terms of pain, dysesthesia, and kinesiophobia in older Japanese adults after lumbar surgery was validated using cross-sectional data. Interventional studies on physical activity based on this model are required to establish the model.

## 1. Introduction

The Japanese population is aging, with the percentage of older adults (≥65 years) at 27.6% in 2017, the highest in the world, and is projected to rise to as high as 38.4% by 2065 [1]. Among the various diseases that occur with aging, osteoarthritis, which causes motor dysfunction, is one of the most common diseases in older adults [2]. Lumbar spinal stenosis (LSS) is another disease caused by the degeneration of the spinal column, which is a motor system. LSS causes not only decreased motor function of the spinal column but also compression of the nerves and blood vessels traveling in the spinal canal, resulting in weakness of leg muscles, pain and dysesthesia (an unpleasant abnormal sensation) in the legs, and neurogenic intermittent claudication [3].

In a review comparing the outcomes of conservative and surgical treatment for patients with LSS and neurogenic intermittent claudication, conservative treatment was observed to have a smaller effect on pain than surgical treatment [4]. Due to the great impact of pain and symptoms in the legs on daily life, such as mobility and activity [5], LSS is considered one of the major conditions for which people over 65 years of age undergo surgery [6]. The outcome of surgical treatment is good [7]; however, it has been revealed that patients complain of mild to moderate postoperative pain [8]. Therefore, postoperative older adults with LSS are not yet necessarily cured. Therefore, there is a need for deeper understanding of the relationship between pain, dysesthesia, and health-related quality of life (HRQOL) to develop a postoperative support.

A review of previous research on the relationship between pain and dysesthesia and HRQOL demonstrated that pain and dysesthesia directly affect HRQOL and that cognitions of pain act as mediating variables that affect HRQOL. Such mediation by cognitions of pain is consistent with the framework of the fear-avoidance model (FAM) [9]. Various concepts have been proposed as cognitions of pain, such as kinesiophobia, catastrophizing [10, 11], and self-efficacy [12, 13]. Among them, kinesiophobia has been the main focus of many studies, and a systematic review revealed that it is associated with pain intensity and HRQOL [14]. A two-factor model, somatic focus (SF) and activity avoidance (AA), is proposed for kinesiophobia [15]. Both factors are associated with HRQOL in older adults after lumbar surgery [16]. Thus, kinesiophobia is a key factor in improving HRQOL because it bridges the relationship between pain and HRQOL.

Treatments for chronic pain using physical activities have been attempted [17]. It has been reported that physical activities have an analgesic effect (so-called exercise-induced hypoalgesia, EIH) [18, 19]. Furthermore, there is evidence to support the modification of cognitions of pain (kinesiophobia and catastrophizing) through behavioral techniques, including physical activities [20, 21]. Hence, the authors hypothesized that physical activities would cause reduction in the frequency of pain and dysesthesia and reduction in kinesiophobia, which in turn improve HRQOL. However, no reports have tested a model that adds physical activities to the relationship between pain and dysesthesia, kinesiophobia, and HRQOL.

Therefore, this study aimed to assess the validity of our hypothesized model, predicting that physical activity reduces kinesiophobia along with pain and dysesthesia and consequently improves HRQOL in older adults who underwent surgery for LSS more than 1 year previously. We used structural equation modeling to examine whether our hypothetical model fits the cross-sectional data.

## 2. Materials and Methods

### 2.1. Research Design and Ethical Considerations

This was a cross-sectional, observational study and extension of a previously published study series [16]. In the previous study in the series, we focused on clarifying the psychometric properties of the Tampa scale for kinesiophobia (TSK) used in this study. The hypothesized model for this study was based on the findings of the previous study.

A questionnaire-based survey was conducted between October 2019 and August 2020. The respondents were required to mention their names in the questionnaire to combine the questionnaire with the medical records at the medical facilities.

The study protocol was approved by the Ethical Review Committees of Sapporo Maruyama Orthopedic Hospital and Harunaso Hospital (approval number: 000025 and 190105, respectively).

The research description and consent forms were distributed to the participants, and informed consent was obtained from all participants. In addition, a consent withdrawal form was provided to the participants to ensure that they had the option to withdraw from the study after participation. If a withdrawal form was submitted, data obtained from the respondents were deleted.

### 2.2. Participants

A total of 422 individuals aged ≥65 years underwent surgery for LSS at Sapporo Maruyama Orthopedic Hospital and Harunaso Hospital at least 1 year ago, and whose current address was known were included. In this study, lumbar disc herniation and degenerative lumbar spondylolisthesis were considered LSS types [22].

### 2.3. Survey Items

#### 2.3.1. Clinical Demographic Information

Age, sex, date of surgery, and surgical technique (decompression fixation or decompression) were obtained from the medical records. The number of postoperative days was defined as the number of days between the date of the surgery and the postmark on the return envelope.

#### 2.3.2. Physical Activities

Using a 5-point Likert scale, participants were asked how often they performed five types of physical activities (walking, stretching, and light-intensity exercises (S/LIE), muscle strength exercises (MSE), maintenance tasks of the house and garden, including kitchen garden (MTH/G), and paid works) [23] often performed by older adults after lumbar surgery: 1 was “never,” 2 was “irregularly,” 3 was “once or twice a week,” 4 was “three or four times a week,” and 5 was “five or more days a week.”

#### 2.3.3. Pain and Dysesthesia

The average intensities of lower back pain (LBP), leg pain (LP), and leg dysesthesia (LD) that continued postoperatively in the last month were assessed using an 11-point numerical rating scale (NRS) (0 points for “no pain or dysesthesia” and 10 points for “intolerable pain or dysesthesia”). The validity of the NRS as a scale for assessing pain intensity has been validated [24].

#### 2.3.4. Kinesiophobia

The Japanese version of the original TSK [25] was used [26]. The internal consistency, re-test reliability, and validity of the TSK have already been confirmed [27, 28]. In this study, the subdomains of the TSK, namely, SF and AA, were employed: the SF was based on the total scores (7–28 points) of items 2, 3, 5, 6, 7, 9, and 11, and the AA was based on the total scores (6–24 points) of items 10, 13, 14, 15, 16, and 17 (item 16 was reversed scores) [16]. The SF refers to the tendency to focus awareness on physical symptoms that are not related to health conditions [29], with higher scores indicating a stronger tendency in each domain.

#### 2.3.5. HRQOL

The self-administered EuroQol-5 Dimension-5 Levels (EQ-5D) was used to assess HRQOL [30]. Validation studies have already been conducted in stroke patients [31] and hip and knee osteoarthritis patients [32].

The EQ-5D consists of five questions related to mobility, self-care, daily living, pain/discomfort, and anxiety/depression, and the participants answered each question on a five-point scale based on the degree of disability and symptoms. In this study, the participants were asked to look back over the last month and answer questions. The EQ-5D index value [33] was calculated based on the answers to each question.

### 2.4. Analysis Procedure

First, missing values were complemented by stochastic regression imputation. The datasets before and after the completion of missing values were compared using the Mann–Whitney *U* test. In addition, descriptive statistics were calculated for each item.

**Figure 1:**
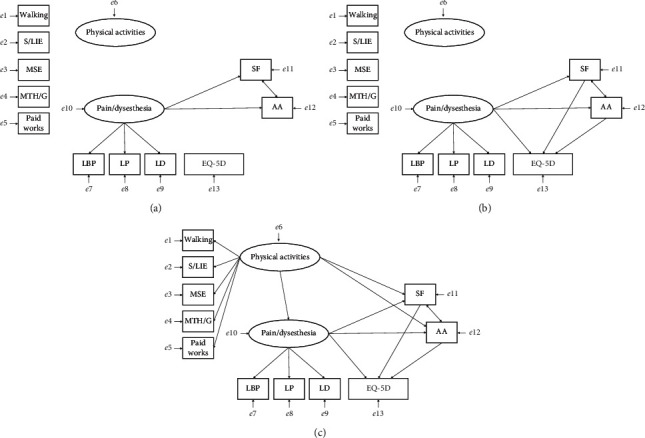
Process of constructing a hypothetical model of the relationship between physical activities and pain/dysesthesia and kinesiophobia. S/LIE: stretching and light-intensity exercises; MSE: muscle strength exercises; MTH/G: maintenance tasks of the house and garden including kitchen garden; SF, somatic focus; AA, activity avoidance; EQ-5D, EuroQol 5-dimension.

A hypothesis model based on FAM was developed (see the following for details):

- Pain/dysesthesia was created as a latent variable, and the NRS scores for LBP, LP, and LD were set as their components. Pain/dysesthesia, a latent variable, drew a path toward SF and AA, which are subconcepts of kinesiophobia. The covariance was set for SF and AA (Figure 1(a)).
- The EQ-5D received a direct path from pain/dysesthesia and an indirect path from SF and AA, respectively. Therefore, pain/dysesthesia directly affects the EQ-5D and indirectly via kinesiophobia (Figure 1(b)).
- Physical activity, a latent variable consisting of walking, S/LIE, MSE, MTH/G, and paid work, was created. Physical activity added a path toward pain/dysesthesia, as well as SF and AA (Figure 1(c)).

The aforementioned hypothetical model was tested for validity using structural equation modeling. The weighted least squares mean variance (WLSMV) estimation developed for ordinal scales was used [34, 35]. The variance of the latent variables was fixed at 1. The comparative fit index (CFI), Tucker–Lewis index (TLI), root mean square error of approximation (RMSEA), and standardized root mean square residual (SRMR) were used to check the goodness-of-fit of the model. CFI and TLI >0.90 were considered to be “adequate,” and >0.95 were considered to be a “good fit.” RMSEA and SRMR were rated as close fit for values <0.05, fair fit for values between 0.05 and 0.08, and a poor fit for values >0.10 [36, 37]. Model modifications were considered if the modification indices were >3.84 [38].

*R* (ver. 4.1.2, *R* Foundation, Vienna, Austria) was used for all statistical analyses, and a risk rate of 5% was set as the level of statistical significance.

## 3. Results

### 3.1. Clinical Demographic Variables of Respondents

**Figure 2:**
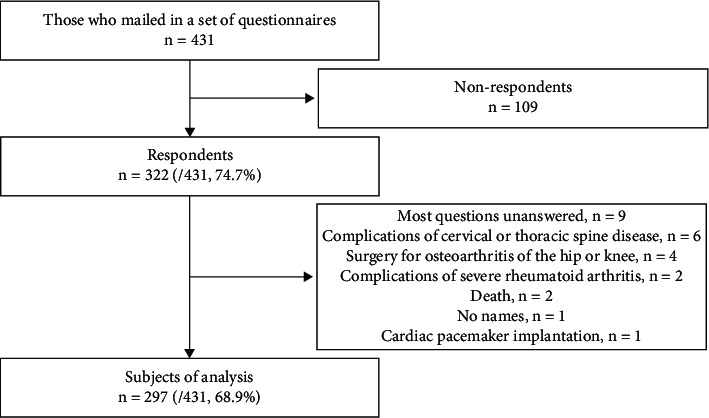
Flowchart of extracting participants for analysis. A total of 431 older adults who had undergone surgery for lumbar spinal stenosis were included in the study and 322 responded (74.7%). Ultimately, data from 297 individuals (68.9%) were analyzed.

A complete set of questionnaires was mailed to 431 participants who met the inclusion criteria. A total of 322 respondents (74.7%) responded. Of these, 25 (nine who did not answer most of the questions, six with cervical or thoracic spine disease, four with osteoarthritis of the hip or knee, two with severe rheumatoid arthritis, two who died, one who was unnamed, and one with a cardiac pacemaker implant) were excluded, resulting in a total of 297 participants (median age 72 years, quartile deviation 3.0 years, range 65–91 years; 158 men, 139 women) included in the analysis (68.9%) (Figure 2). The median number of postoperative days in the analysis group was 606 (quartile deviation 195.0 days, range 373–1531 days), with 94 (31.7%) patients undergoing decompression only and 203 (68.4%) undergoing decompression and fixation.

### 3.2. Completing Missing Values and Descriptive Statistics of Survey Items

Since the number of survey items per person was 30 (NRS: 3, EQ-5D: 5, TSK: 17, and physical activity: 5), the total number of questionnaire items collected from 297 people was 8910. Among these, there were 318 with missing values (3.6%). Missing values were obtained using MICE. When the original dataset with missing values was compared with the complete dataset after completion, no significant difference was observed.

The descriptive statistics of the survey items are summarized in Table 1.

### 3.3. Goodness-of-Fit of the Hypothetical Model and the Association between Observed and Latent Variables

**Figure 3:**
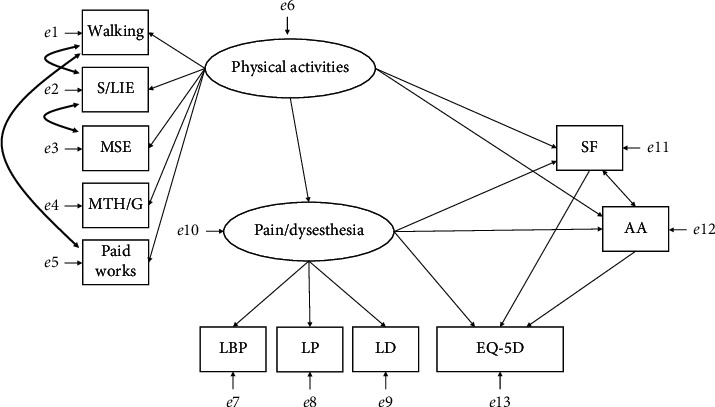
The model revised with reference to modified indices. The thick two-way arrows are the covariances added to the hypothetical model. S/LIE: stretching and light-intensity exercises; MSE: muscle strength exercises; MTH/G: maintenance tasks of the house and garden including kitchen garden; SF, somatic focus; AA, activity avoidance; EQ-5D, EuroQol 5-dimension.

The goodness-of-fit indices obtained from the hypothetical model of this study were 0.840 for CFI, 0.768 for TLI, 0.084 (90% confidence interval 0.067–0.101) for RAMSEA, and 0.072 for SRMR. Because only SRMR revealed acceptable values, three covariances were added between walking, S/LIE, and paid work and between S/LIE and MSE with reference to the modification indices (Figure 3). After modifying the model, the values of the goodness-of-fit indices improved to 0.948 for CFI, 0.919 for TLI, 0.048 (90% confidence interval 0.026–0.069) for RAMSEA, and 0.046 for SRMR. CFI and TLI were judged to be adequate, while RMSEA and SRMR were judged to be a close fit. The modified model estimates are listed in Table 2.

## 4. Discussion

`The purpose of this study was to clarify the clinical significance of maintaining and improving physical activity in older adults who had undergone surgery for LSS more than 1 year earlier. To achieve this goal, we tested whether the model that physical activities can reduce kinesiophobia, as well as pain and dysesthesia, and consequently improve HRQOL, is valid.

### 4.1. Characteristics of the Respondents

Older adults who had undergone surgery for LSS >1 year earlier were included. The median NRS scores for LBP, LP, and LD were low. In contrast, the EQ-5D index values were comparable to the mean of the Japanese individuals aged ≥50 years who complained of nonspecific LBP and were lower than the mean of those without nonspecific LBP [39]. The most frequent physical activities performed by the respondents were walking and MTH/G, which were performed by more than half of the respondents. Few respondents engaged in high-intensity physical activities, such as muscle strength exercises, but many engaged in low-intensity physical activities, such as walking and household activities, irregularly or once or twice a week.

### 4.2. Goodness-of-Fit of the Modified Hypothesized Model

Based on the FAM, pain and dysesthesia directly affected HRQOL and indirectly through the mediation of kinesiophobia [14, 40]. In the present study, we added the paths by which physical activities influence pain, dysesthesia, and kinesiophobia (SF and AA). In the initial hypothetical model constructed in this manner, the goodness-of-fit indices did not meet these criteria.

According to the modification indices, the covariance between walking and S/LIE, walking and paid work, and S/LIE and MSE improved the goodness-of-fit to a level that made the model acceptable. A significant positive covariance was observed between walking and S/LIE, and between S/LIE and MSE. It has been reported that people with personality traits, such as extroversion and conscientiousness, are more active [41], and that the presence or absence of these traits may act as hidden common factors. Otherwise, the covariance between walking and paid work is negative. Retirement is a life event that increases the level of physical activity during leisure time [42], and this suggests that the time spent on paid work and leisure time is inversely related. Thus, the addition of the three covariances is reasonable and acceptable.

### 4.3. Effects of Physical Activities on Pain, Dysesthesia, and Kinesiophobia

As hypothesized in this study, the paths by which physical activity reduced the intensity of pain and dysesthesia and consequently improved HRQOL were significant. This result is consistent with the findings of Nambi et al., who reported that yoga successfully reduced the intensity of chronic LBP and improved HRQOL [43]. As it has been reported that moderate aerobic exercise lasting >10 min can temporarily reduce the intensity of tenderness [44], it is known that physical activities can reduce pain and dysesthesia, which has been termed EIH. EIH is thought to be an analgesic based on a decrease in substance P, which causes hyperalgesia, and an increase in beta-endorphin, an endogenous opioid, and cortisol, an anti-inflammatory agent [45]. This analgesic mechanism may be involved in older adults after lumbar spine surgery.

Furthermore, the path by which physical activity reduces kinesiophobia and consequently improves HRQOL is significant. This suggests that physical activities affect psychological factors in addition to their effects on pain. These paths from physical activity to HRQOL via kinesiophobia are supported by the following two reports. Patients with nonspecific LBP at low levels of physical activity had strong kinesiophobia [46]. Moreover, kinesiophobia was a determinant of HRQOL and pain [47].

When considering two aspects of kinesiophobia, “tendency to focus on physical symptoms” and “avoidance beliefs about physical activities” had a direct effect on HRQOL in our model, while “avoidance beliefs about physical activities” had only an indirect effect on HRQOL in relation to “tendency to focus on physical symptoms.” In a study of women with patellofemoral joint pain, both “avoidance beliefs about physical activities” and “tendency to focus on physical symptoms” affected HRQOL [7]. Factors causing this discrepancy were not clarified. However, items belonging to the domain of “tendency to focus on physical symptoms” may have explained HRQOL better than the “avoidance beliefs about physical activities” in older adults after lumbar surgery because many of the items belonging to the domain of “tendency to focus on physical symptoms” affected HRQOL.

### 4.4. Strengths and Limitations

To the best of our knowledge, this is the first study to validate a model predicting HRQOL improvement due to physical activity in older Japanese adults with pain, dysesthesia, and kinesiophobia who had undergone surgery for LSS. The model is consistent with a report that physical activity improves HRQOL in patients with chronic pain [17] and is novel in that kinesiophobia, as well as pain and dysesthesia, are introduced as intermediate processes. One of the strengths of this study is that the data were collected from multiple institutions. This increased the generalizability of the model proposed in this study.

This study has two main limitations. First, our cross-sectional data in this study may contain recall and dropout biases. Participants were asked to recall their pain and health status in the last month, but it is not guaranteed that participants' self-reports will match their true status. In addition, approximately 30% of the participants were excluded from the analysis. It is possible that the 30% dropout rate may have biased our data. These biases limit the generalizability of the findings in this study. Second, the hypothetical causal model assessed in this study fits well with our cross-sectional data, but causal relationships between physical activity and HRQOL in terms of kinesiophobia, pain, and dysesthesia have not been determined. Moreover, it does not consider factors that may influence pain, kinesiophobia, and physical activities, such as sex, season, or region of residence. The prevalence and severity of chronic pain are reportedly higher in women [48], and sex is a moderator variable in the relationship between pain and kinesiophobia [49]. In addition, the physical activities of older adults differed according to the region of residence (urban vs. rural) [50] and season [51]. In the future, it will be necessary to confirm the causal relationship between physical activity and HRQOL in terms of kinesiophobia, pain, and dysesthesia through interventional studies on physical activities and to verify the robustness of the model of this study by sensitivity analysis.

## 5. Conclusions

We assessed the validity of our hypothesized model that physical activity affects HRQOL in older Japanese adults who underwent surgery for LSS more than 1 year previously. Structural equation modeling using WLSMV, a statistically robust method, revealed that physical activities were significant both in the path of improving HRQOL by reducing the intensity of pain and dysesthesia and improving HRQOL by reducing kinesiophobia. Although the hypothetical causal model was validated in our cross-sectional data, intervention studies on physical activity are required to confirm the causal relationships.

## Figures and Tables

**Table 1:** Descriptive statistics of the assessed items.

Items (range)	Median	Quartile deviation	Minimum-maximum
Pain/dysesthesia			
LBP (0–10 points)	2	1.5	0–10
LP (0–10 points)	1	1.5	0–8
LD (0–10 points)	1	1.5	0–9

HRQOL			
EQ-5D (0–1 points)	0.728	0.096	0.01–1

Kinesiophobia			
SF (7–28 points)	15	2.0	7–26
AA (6–24 points)	15	1.5	6–22

Physical activities			
Walking (1–5 points)	2	1.0	1–5
S/LIE (1–5 points)	1	1.5	1–5
MSE (1–5 points)	1	1.0	1–5
MTH/G (1–5 points)	2	1.0	1–5
Paid works (1–5 points)	1	0.5	1–5

LBP, lower back pain; LP, leg pain; LD, leg dysesthesia; HRQOL, health-related quality of life; EQ-5D: EuroQol 5-dimension, SF, somatic focus; AA, activity avoidance. S/LIE, stretching and light-intensity exercises; MSE, muscle strength exercises; MTH/G, maintenance tasks of the house and garden, including kitchen garden.

**Table 2:** Estimates and standardized estimates obtained by the WLSMV estimation.

	Estimates	*p* values	Standardized estimates
Latent variables			
PA<-			
Walking	**0.619**	**0.000 ** ^**a**^	**0.418**
S/LIE	0.149	0.0270	0.109
MSE	**0.247**	**0.035 ** ^**b**^	**0.191**
MTH/G	**0.571**	**0.000 ** ^**a**^	**0.484**
Paid works	0.170	0.187	0.119
Pain/dysesthesia<-			
LBP	**1.474**	**0.000 ** ^**a**^	**0.798**
LP	**1.634**	**0.000 ** ^**a**^	**0.816**
LD	**1.539**	**0.000 ** ^**a**^	**0.687**

Regressions			
AA<-			
Pain/dysesthesia	−0.03s8	0.878	−0.014
PA	**−1.030**	**0.003 ** ^**a**^	**−0.355**
SF<-			
Pain/dysesthesia	**1.082**	**0.001 ** ^**a**^	**0.309**
PA	**−1.157**	**0.005 ** ^**a**^	**−0.301**
Pain/dysesthesia<-			
PA	**−0.445**	**0.002 ** ^**a**^	**−0.406**
EQ-5D<-			
Pain/dysesthesia	**−0.110**	**0.000 ** ^**a**^	**−0.684**
AA	−0.001	0.800	−0.011
SF	**−0.010**	**0.000 ** ^**a**^	**−0.218**

Covariances			
AA<->			
SF	**2.429**	**0.002 ** ^**a**^	**0.271**
Walking<->			
S/LIE	**0.574**	**0.000 ** ^**a**^	**0.313**
Paid works	**−0.518**	**0.000 ** ^**a**^	**−0.270**
S/LIE <->			
MSE	**0.809**	**0.000 ** ^**a**^	**0.468**

*n* = 297. ^a^:*p* < 0.01; ^b^:*p* < 0.05. <- represents regressions and <-> represents covariances. WLSMV, weighted least squares mean variance; PA, physical activity; S/LIE, stretching and light-intensity exercises; MSE, muscle strength exercises; MTH/G, maintenance tasks of the house and garden, including kitchen garden; SF, somatic focus; AA, activity avoidance; EQ-5D, EuroQol 5-dimension.

## Data Availability

The data that support the findings of this study are available from the corresponding author, Daisuke Higuchi, upon reasonable request.
